# No Significant Effects of Cellphone Electromagnetic Radiation on Mice Memory or Anxiety: Some Mixed Effects on Traumatic Brain Injured Mice

**DOI:** 10.1089/neur.2021.0009

**Published:** 2021-08-17

**Authors:** Doaa Qubty, Shaul Schreiber, Vardit Rubovitch, Amir Boag, Chaim G. Pick

**Affiliations:** ^1^ Department of Anatomy and Anthropology, Sackler School of Medicine, Tel Aviv University, Tel Aviv, Israel.; ^2^ Department of Psychiatry, Tel Aviv Sourasky Medical Center, Tel Aviv, Israel.; ^3^ Sagol School of Neuroscience, Tel Aviv University, Tel Aviv, Israel.; ^4^ School of Electrical Engineering, Tel Aviv University, Tel Aviv, Israel.; ^5^ The Dr. Miriam and Sheldon G. Adelson Center for the Biology of Addictive Diseases, Tel Aviv University, Tel Aviv, Israel.; ^6^ Sylvan Adams Sports Institute, Tel Aviv University, Tel Aviv, Israel.

**Keywords:** cellphone, electromagnetic radiation, mice, traumatic brain injury

## Abstract

Current literature details an array of contradictory results regarding the effect of radiofrequency electromagnetic radiation (RF-EMR) on health, both in humans and in animal models. The present study was designed to ascertain the conflicting data published regarding the possible impact of cellular exposure (radiation) on male and female mice as far as spatial memory, anxiety, and general well-being is concerned. To increase the likelihood of identifying possible “subtle” effects, we chose to test it in already cognitively impaired (following mild traumatic brain injury; mTBI) mice. Exposure to cellular radiation by itself had no significant impact on anxiety levels or spatial/visual memory in mice. When examining the dual impact of mTBI and cellular radiation on anxiety, no differences were found in the anxiety-like behavior as seen at the elevated plus maze (EPM). When exposed to both mTBI and cellular radiation, our results show improvement of visual memory impairment in both female and male mice, but worsening of the spatial memory of female mice. These results do not allow for a decisive conclusion regarding the possible hazards of cellular radiation on brain function in mice, and the mTBI did not facilitate identification of subtle effects by augmenting them.

## Introduction

### Cell phone electromagnetic radiation

It is estimated that as of the end of 2020, about 50% of the world's population relies on cell phones for communication.^1^ The exponential growth of personal telecommunication devices, like the global system for mobile communication (GSM) cell phone, has been an issue of endless discussions and extended research efforts worldwide both in humans^2–4^ and in animal models,^5,6^ with conflicting results in rodents and controversy to the existence or not of cognitive deficits, even though no “smoking-gun” study has been published to date. The public health concern regarding the potential risk of chronic exposure to the low levels of radiofrequency and microwave (RF/MW) or electromagnetic radiation (EMR) that is emitted by the phone antenna, derives from the use of the device in close proximity to the user's head.^7^

Cell phone frequencies vary according to the system of use, either 900 or 1800 (MHz) or 2200 MHz according to Universal Mobile Telecommunications System (UMTS). The limit of mobile phone radiation exposure level set in the United States and Europe is 1.6 W/kg and 2 W/kg, respectively.^8^ Generally, people receive exposures close to these limits due to the use of handheld mobile phones and cordless phones in various positions with respect to the body. With the transmitting devices in these positions during use, electromagnetic fields in the frequency range of 40 MHz to 6 GHz often penetrate deep into the tissue, causing an increase in the random molecular motion.^7^

Radio frequency radiation (RFR) of mobile phones raises concerns about possible implications to human health.^4,9^ It has been widely investigated using an epidemiological approach where several reports showed no association between cell phone use and brain tumors, whereas other reports came to the opposite conclusion. Headache has been reported among cell phone users compared with non-users.^10^

In spite of previous studies, knowledge about the possible adverse effects of RF/MW radiation on human health, or the biological responses to RF/MW radiation exposure is still limited.

### Traumatic brain injury

Traumatic brain injury (TBI) affects a significant part of the younger population and is considered the most common neurological disorder among people less than 50 years of age.^11^ TBI is caused when the head is impacted by force. It is often associated with falls, particularly among the elderly, injuries due to sport activity, and military operations among young people.^11,12^ Patients with TBI may suffer various degrees of short-term and long-term cognitive, behavioral, and emotional impairment, not always in direct concordance with the severity of the injury.

There is evidence that TBI (or repeated episodes of mTBI in the case of chronic traumatic encephalopathy, CTE) is an important risk factor for the development of neurodegenerative diseases, such as Alzheimer's and Parkinson's disease.^13–15^ TBI is classified into three severity levels: mild, moderate, and severe. Severity of the injury is classified according to the Glasgow Coma Scale (GCS) when a patient is admitted to the hospital. A mild injury is assessed as a GCS score of 13–15, moderate injury as a GCS score of 9–12, and severe injury as a GCS score below 9.^16^ To date, there is still no proven treatment for prevention or reduction of damage following a TBI, hence it is quite important to understand the pathological mechanisms that occur after TBI to develop and explore appropriate treatment strategies.^17,18^

### Mild traumatic brain injury

More than 90% of head injury cases are defined as mTBI.^16^ In contrast to severe brain injury, which is diagnosed by analyzing direct brain damage, blood–brain barrier (BBB) rupture, and edema, mTBI diagnosis is more difficult, as routine (anatomical) imaging tests (computed tomography [CT], magnetic resonance imaging [MRI]) fail to show structural changes in the brain,^19^ and functional brain imaging (functional MRI [fMRI], positron emission tomography [PET]-MRI, single photon computed emission tomography [SPECT]) are seldom used for clinical evaluation of these patients. However, some survivors of mTBI suffer from cognitive and emotional difficulties over time, such as concentration difficulties, memory and sleep problems, depression, and anxiety.^20,21^ In most patients with mTBI, the symptoms subside within a year of the impact, but many survivors suffer from persistent neurocognitive effects. This is defined as post-concussive syndrome.^22,23^ Recently, several epidemiological studies have reported that mTBI is also considered an important risk factor for neurodegenerative diseases, such as Alzheimer's and Parkinson's disease.^14,24^

The cascade of pathological events that occurs after mTBI is a complex process, and can be separated into early damage and secondary damage. The early damage occurs at the time of injury as an immediate and direct result of the external energy exerted by compression and cutting forces on the neural tissue and blood vessels. The initial damage may directly harm the brain tissue, axonal injury (shearing of the axons, apoptosis of synapses), and transient infiltration into the BBB. This damage can start a cascade of pathological events that will lead to additional brain damage, defined as secondary. Secondary damage may manifest several days to weeks or even months after the initial injury, and it may include brain edema, inflammatory responses with a notable release of pro-inflammatory cytokines, increased production of free radicals, release of glutamate from the injured tissue leading to glutamate-induced toxicity, and various degrees of DNA damage. When the extent of the damage is such that the neurons fail to overcome it, secondary damage will eventually cause apoptosis.^25,26^ Some studies suggest that secondary damage may be limited or even reversible by compounds that interfere with one or more of the pathological pathways that cause it.^16,25^

To investigate the consequences of TBI, several models of head injuries have been developed for laboratory animals. The main models used to induce TBI are lateral fluid-percussion (LFP), which imposes a diffuse injury on the exposed dura by rapid injection of liquid (usually saline) that spreads to the epidural space,^27^ controlled cortical impact (CCI), which is a penetrating method in which a rigid body causes direct mechanical damage to the dura by air pressure, which serves as the source of mechanical force,^28^ and the weight-drop model in which injury is induced through the free fall of a weight directly onto a closed or open skull. The severity of the injury is determined by the height of the tube and the weight of the pellet.^12,29,30^

Our lab uses a closed-head weight-drop model to obtain an mTBI; this model simulates injuries to people producing memory and learning difficulties in the absence of anatomical damage seen in the brain.^12^ Previous studies in our laboratory have shown that after experimental mTBI in mice, there are behavioral and cognitive changes 7 and 30 days after injury. Various tests were used, such as the Morris water maze; the passive avoidance test (which examines management functions); Y-maze; spatial memory recognition; and visual memory inspection, among others.^12,19,31^

The present study was designed to provide new insight into the conflicting data published regarding the possible impact of cellular exposure (radiation) on male and female mice as far as their spatial memory, anxiety, and general well-being are concerned, and to increase the likelihood of identifying possible “subtle” effects, we chose to test it in already impaired (following mTBI) mice.

## Methods

### Mice

ICR male and female mice 6–8 weeks of age, within the average weight of 30–40 g were studied. Mice were housed in groups of five in each cage, in a soundproof room with 12-h light/dark cycles and at a constant room temperature of 22°C. The animals were given 2 to 3 days of habituation upon their arrival at the animal facility. Purina rodent chow was given *ad labium* during the entire experimental period. We had four groups: 1) control group; 2) TBI mice; 3) mice exposed only to cellular radiation; and 4) mice exposed to both cellular radiation and a TBI. The minimum number of mice was used to facilitate results (*n* = 134: 40 female/94 male).

All procedures were in line with the Guidelines for Animal Experimentation of the National Institutes of Health (DHEW publication 85-23, revised, 1995), and were approved by the Sackler Faculty of Medicine Ethics Committee (01-17-045).

### Cell phone radiation exposure

For the radiation exposure, two plastic cages of mice (each cage 12 cm height, 21.5 cm width, 32 cm length) were randomly picked; both were exposed to cell phone radiation for a week and mice in only one of them had TBI induction following that week. The two groups continued for additional 5 weeks of exposure until the behavioral tests. To assure that the radiation would not reach any other cage and would affect only the study animals, during the whole period of the experiment these cages were kept inside a steel box (30.8 cm height, 45.6 cm width, 80.6 cm length). Each steel box had several holes to allow a consistent flow of air and to maintain stable temperature. The control group cages were kept in the same manner: two plastic cages in one steel box. The box with the cages was placed in a special soundproof room. For the entire duration of the experiment, the mice were exposed to phone radiation through a video call via Skype or FaceTime, using an iPhone 7 that was placed approximately 10 cm above the mice. To maximize the effect of the radiation (electric field intensity 20 ± 5 V/m, standard deviation [SD]), we exposed the mice to 4–5 h of video calling per day for 6 weeks. The phones were kept on silent mode and an automatic answering system was used to reduce opening the cages and potentially cause a disruptive contact with the mice. Because the mice were kept in a room with 12 h of light followed by 12 h of darkness, we only made the calls during the day to minimize light exposure and disrupt the normal circadian rhythms of the mice. The cellphone display was kept facing in the opposite direction of the mice to further reduce any potential exposure.

**Fig 1.**
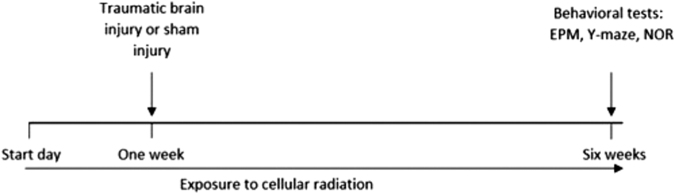
Experiment timeline; mice were exposed to cellular radiation for a week before TBI induction. After 5 more weeks, the mice went through a series of behavioral tests including the EPM, Y-maze, and NOR. EPM, elevated plus maze; NOR, novel object recognition; TBI, traumatic brain injury.

On the seventh week of the exposure the animals went through the behavioral tests. All experiments were conducted in blind fashion (Fig. 1).

### Traumatic brain injury

Brain injury was induced using a weight-drop head trauma device, as previously described.^32^ The device consists of a metal pipe with an internal diameter of 13 mm and a length of 80 cm. Each mouse was lightly anesthetized, by isoflurane inhalation, before the injury induction and placed immediately below the device on top of a sponge to fix its head. A 30-g weight was released at the top of the tube for a free fall along its length to hit the right temporal lobe of the mouse's brain between the eye and the ear. Immediately after the injuries were induced, the mice were returned to their home cages for recovery and follow-up.

Control mice were treated similarly to mTBI-challenged mice; they were anesthetized with isoflurane and were aligned under the weight-drop device, but were not exposed to the injury, to assure that the anesthesia did not induce any deficits. Then all the animals were returned to their cages for recovery. Control and mTBI mice were indistinguishable following their recovery from the procedure. Body temperature has been shown in our previous studies with this procedure to be maintained, and the selection of animal number for individual studies (whether behavioral, immunohistochemical, or biochemical) was determined from the variance in the data of our prior studies.

### Behavioral tests

“Basic well-being” is a concept that underlies combined health and wellness parameters. Four parameters were evaluated to define the mice's basic well-being: rectal temperature, motor skills, pain threshold, and anxiety levels.^33^

### Elevated plus maze paradigm

The elevated plus maze (EPM) test is used to assess levels of anxiety and fear among rodents as previously described.^34^ The test relies on the innate conflict between approaching and exploring versus avoiding potentially dangerous areas.^35^ The bright and exposed regions of the EPM represent the “dangerous” areas of the mazes, whereas the darkened and enclosed regions are perceived as safer. The maze is built from black Plexiglas. It sits 50 cm above the ground. The maze is in the shape of a plus sign, with two arms perpendicular to one another. The first arm of the plus sign (30 × 5 × 1 cm) is a closed arm with walls, whereas the other arm (15 × 5 × 30 cm) is an open one. On the day of the experiment, each mouse was inserted into the center of the maze facing one of the non-walled arms, and it was allowed 5 min to explore the arms. Time spent in the open arms during the 5 min was recorded. Longer duration of time spent within the open arms has been associated with lower anxiety levels,^36^ whereas higher number of entries to the open/closed arms is indicative to higher locomotor activity. The maze was cleaned after each session using a 70% ethanol solution and dried to prevent any olfactory recognition.

### Novel object recognition task

The novel object recognition (NOR) task was chosen to assess the recognition and visual memory of the mice, as previously described.^33^ This task is based on the innate tendency of rodents to explore new objects within their environment.^37^ The use of this natural tendency allows one to determine whether a mouse can discriminate between a familiar and a novel object. Mice were individually habituated to an open field Plexiglas arena (59 × 59 × 20 cm) for a period of 5 min. Twenty-four hours later, in the acquisition phase, two identical objects (A and B) were placed in a symmetrical position within the arena. The objects were sufficiently large to ensure that the mice could neither move nor climb over them.

During the memory recognition assessment phase that was assessed 24 h thereafter, one of the objects (A or B) was randomly replaced by a novel one (C), and the mouse exploratory behavior was analyzed over a 5-min period. Exploration of an object was defined as rearing on the object, sniffing it at a distance of less than 2 cm, and/or touching it with the nose. Successful recognition was represented by preferential exploration of the novel object over the familiar object. The time spent by each mouse exploring the novel object over the familiar object was recorded and used to generate a preference index. A discrimination preference index was calculated as follows: (time spent near the new object minus time spent near the old object)/(time spent near the new object plus time spent near the old object).^37^ After each session, the objects and arena were thoroughly cleaned with 70% ethanol to prevent odor recognition.

### Y-maze pardigm

The Y-maze paradigm was used to evaluate spontaneous exploration, responsiveness to novel environments, and spatial memory function, as previously described.^38^ The apparatus used for the Y-maze study was a three-armed black Plexiglas maze with each arm separated by 120 degrees. Each arm measured 8 × 30 × 15 cm and was distinguishable only by spatial cues placed within (i.e., a triangle, a square, or a circle). The start point is from the same arm for all mice. Each mouse was placed into the Y-maze environment on two occasions separated by a 2-min interval, during which the mouse was returned to its home cage. In the first 5-min trial, one of the two arms was randomly blocked. In the second 2-min trial, all arms were open for exploration, and the total amount of time during which the mouse explored each arm was measured. A discrimination preference index was calculated as follows: (time in new arm – time in familiar arm)/(time in new arm + time in familiar arm).^37^

### Statistical analysis

Data are presented as mean ± standard error of the mean (SEM) and analyzed by Prism or SPSS V 19 software. One-way analysis of variance (ANOVA) tests were used for comparison of multiple samples, followed by Dunnett's post-test post hoc tests. In addition, for behavioral experiments Fisher's least significant difference (LSD) post hoc analysis was used: *p ≤ 0.05, **p ≤ 0.01, ***p ≤ 0.001.

## Results

### Behavioral assessments

#### Anxiety and well-being

**Fig 2.**
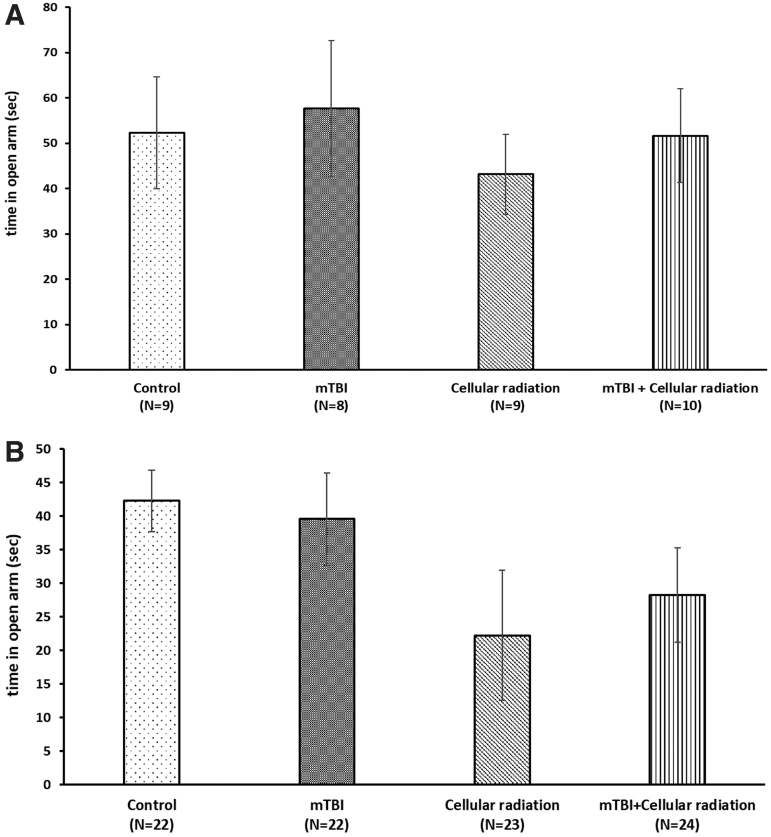
Neither mTBI induction nor cellular exposure affect anxiety-like behavior in both female **(A)** and male mice **(B)**, as was evaluated in the elevated plus maze paradigm (Fig. 1). All groups of mice spent equivalent time in the open arm of the elevated plus maze at 5 weeks post-mTBI. These results indicate that cellular radiation and mTBI do not increase anxiety. The results are shown as mean ± standard error. mTBI, mild traumatic brain injury.

Anxiety-like behavior following mTBI was assessed using the EPM paradigm. Time spent in the open arm of the maze and the number of entries to each arm were recorded. Female/male mice were examined separately at 5 weeks post-mTBI or post-sham injury, with or without exposure to cellular radiation. All groups spent approximately equal time in the open arm of the maze and could not be differentiated from one another, indicating that cellular radiation and mTBI does not increase anxiety-like behavior (Fig. 2 A,B). Basic well-being of mice was not altered throughout the experiments (data not shown).

#### The Y-maze test

**Fig 3.**
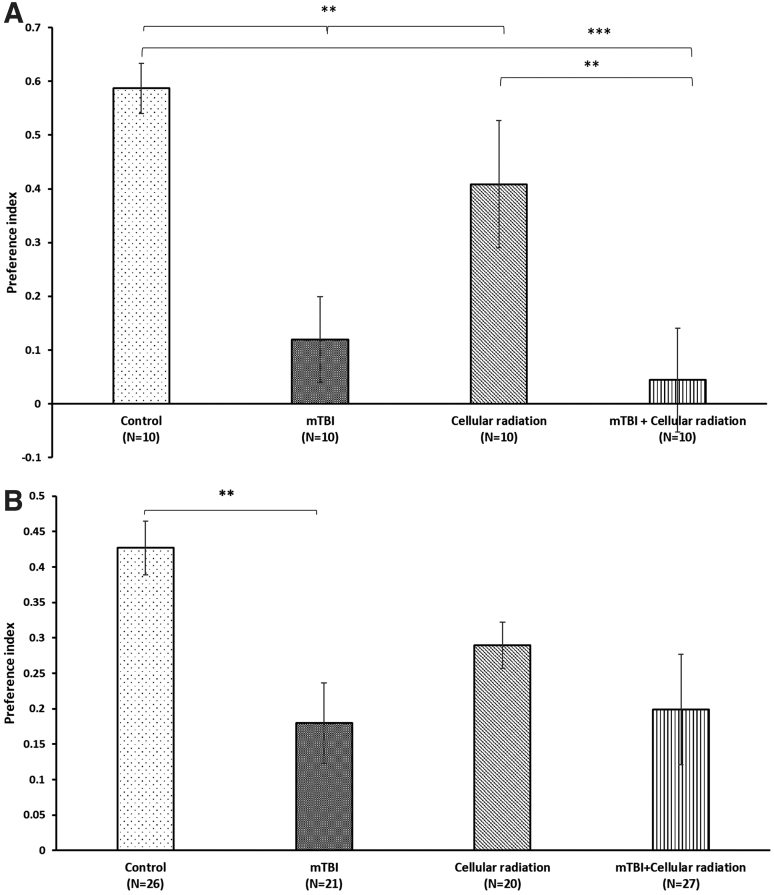
**(A)** In female mice, exposure to cellular radiation post-mTBI induced significant spatial memory deficits compared with the control and cellular radiation group (***p < 0.001, **p < 0.01). mTBI mice demonstrate a deficit in spatial memory compared with control and cellular radiation groups (**p < 0.01) as assessed in the Y-maze paradigm. **(B)** In male mice, mTBI mice demonstrate a deficit in spatial memory compared with control group (**p < 0.01) as assessed in the Y-maze paradigm. A preference index is used to represent the relative time that mice spent exploring the novel arm compared with the old arm, which reflects spatial memory. One-way ANOVA revealed that both female and male mTBI mice had a deficit in visual memory compared with control groups. (F [3, 36] = 8.041, *p* = 0.000 Fisher's LSD post hoc, *p* < 0.001, *n* = 10), (F [1, 46] = 9.042, *p* = 0.001 Fisher's LSD post hoc, *p* < 0.01, *n* = 20–27). Values are presented as mean ± SEM, *p < 0.05; **p < 0.01; and ***p < 0.001. ANOVA, analysis of variance; LSD, least significant difference; mTBI, mild traumatic brain injury; SEM, standard error of the mean.

This test was performed 5 weeks following mTBI to assess spatial-memory; one-way ANOVA revealed that mTBI animals had a deficit in spatial memory compared with control groups in both females and males (Fig. 3 A,B). Exposure to cellular radiation alone had no impact on spatial memory in both female and male mice compared with control group (Fig. 3 A,B). In female mice, mTBI animals had a significant deficit compared with the control group and cellular radiation group, whereas those that were exposed to both cellular radiation and TBI were the most adversely impacted compared with the control and cellular radiation group (Fig. 3A). In contrast to female mice, male mice exposed to cellular radiation did not show a significant deficit in spatial memory following mTBI.

#### The NOR test

**Fig 4.**
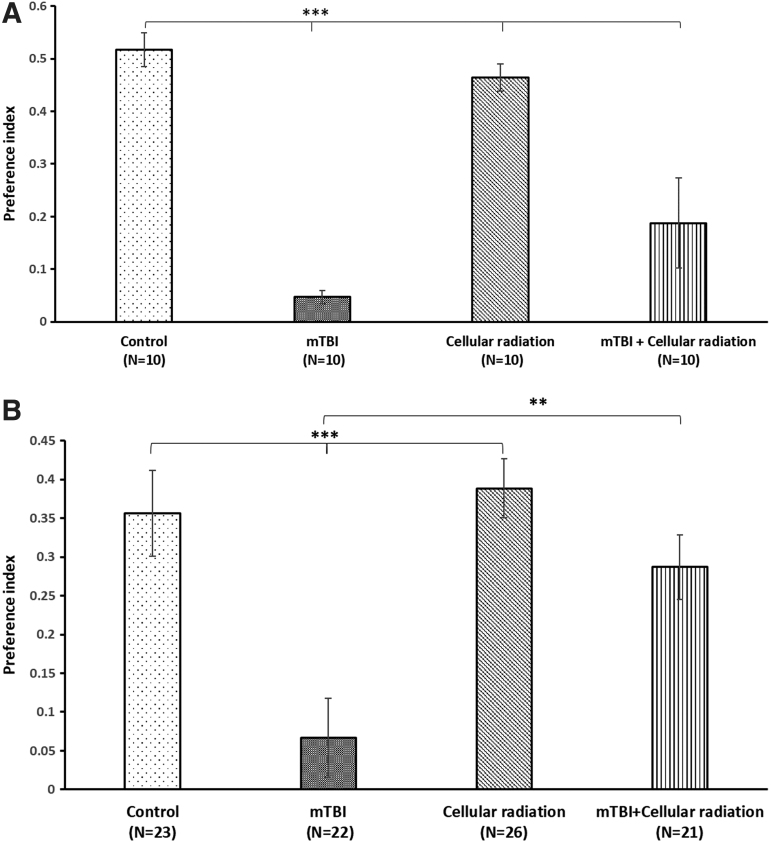
**(A)** Exposure to cellular radiation in female mice mitigated the visual memory deficits in mice post-mTBI, as assessed in the novel object recognition paradigm (***p < 0.001). **(B)** In male mice, exposure to cellular radiation improved the visual memory deficits in mice post-mTBI (**p < 0.01), as assessed in the novel object recognition paradigm. A preference index is used to represent the relative time that mice spent exploring the novel object compared with the familiar object, which reflects visual memory. (F [3, 36] = 11.025, *p* = 0.000 Fisher's LSD post hoc, *p* < 0.001, *n* = 8–10), (F [3, 93] = 7.951, *p* = 0.000 Fisher's LSD post hoc, *p* < 0.001, *n* = 21–26). Values are presented as mean ± SEM, *p < 0.05; **p < 0.01; ***p < 0.001. LSD, least significant difference; mTBI, mild traumatic brain injury; SEM, standard error of the mean.

This test was performed 5 weeks following mTBI. One-way ANOVA revealed that mTBI animals had a deficit in visual memory compared with the control groups in both females and males (Fig. 4A,B). Exposure to cellular radiation had no impact on visual memory in both female and male mice compared with control group (Fig. 4 A,B). Surprisingly, exposure to cellular radiation led to a significant improvement in the visual memory deficits of female and male mice post-mTBI compared with the mTBI mice that were not exposed to radiation (Fig. 4A,B), as assessed by the NOR paradigm.

In summary, we examined the dual impact of TBI and cellular radiation on anxiety, and spatial and visual memory. No differences were found in the anxiety-like behavior as seen at the EPM. In addition, our results show that mTBI by itself impaired spatial and visual memory in both female and male mice. The results also show that exposure to cellular radiation by itself had no significant impact on anxiety levels or spatial/visual memory in mice. When exposed to both mTBI and cellular radiation, our results show improvement of visual memory impairment in both female and male mice. However, the spatial memory of female mice was worsened by the combination of cellular radiation and mTBI.

## Discussion

In the present study we tried to resolve the controversies regarding the possible impact of cellular radiation on several brain functions (i.e., visual memory, spatial memory, anxiety) and general well-being, in female and in male mice. To enhance possible subtle effects of the cellular radiation, we chose to study it mTBI injured mice, in which the tested paradigms have already been assessed and documented.

As shown in previous studies, mTBI impaired spatial and visual memory in both female and male mice but had no effects on anxiety-like behavior evaluated with the EPM. Exposure to cellular radiation by itself had no significant impact on anxiety levels or spatial or visual memory in the control group of mice. When mTBI mice were exposed to cellular radiation, our results show improvement of visual memory impairment in both female and male mice. However, although exposure to cellular radiation of male mTBI mice did not alter the degree of impairment of their spatial memory, the spatial memory of female mice was worsened by the combination of cellular radiation and mTBI.

Tafakori and colleagues^39^ investigated the effects of short-term (1 week) or long-term (4 week) 3 h a day exposure to mobile EMR on the medial prefrontal cortex of rats using the T-maze task. The 1-week (short-term) exposure caused a temporary distinct increment in T-maze working memory task completion time (which returned to normal following a 1-week rest). As for the long-term (4-week) exposure, the same effect was still evident after the second week, but it decreased as exposure continued, until returning to baseline. However, both short-time and long-time exposure to mobile EMR had a negative effect on task accuracy, as a significant reduction in the percentage of correctly performed task was noted in both groups.^39^

Narayanan and colleagues^4^ reviewed a series of studies of RF-EMR-induced behavioral changes in rodents, and suggested many possible mechanisms to explain the changes found. The suggested underlying mechanisms include structural changes in the brain (altered BBB integrity; changes in the cytoarchitecture of the hippocampal formation, the amygdala, the cerebral cortex, or the cerebellum—or a combination of these), effects on glial cells, modulatory impact on various neurotransmitter levels in the brain, possible activation of apoptotic pathway, effects on DNA, and different combinations of these and other mechanisms.^4^

In their systematic review of the possible effects of exposure to low-level RF fields on cognitive behavior (spatial learning and place memory) of laboratory animals (mice, rats, transgenic mice, migratory birds), Sienkiewicz and van Rongen^40^ conclude that “it is not yet possible to give an unequivocal answer to the question.” They further add that some studies reported an adverse effect on spatial learning and memory, whereas others have not reported an effect, and a few others yet reported an improvement in performance—and therefore they suggest that additional basic research is required.^41^ In addition, to avoid exacerbation of the situation in which a plethora of models and protocols complicates the possibility to reach a sustainable result, they suggest the use of a single animal model with standardized exposure and testing protocols.

In this study we utilized a well-established model to test mice following TBI. We created an environment for the mice that resembles, as much as possible, what humans would experience in real life, using EMR from a real mobile phone and exposing the mice to the radiation on a daily basis. We also focused on young mice, aged 6–8 weeks, and continued the exposure for a period of 6 weeks until their age reached 12–14 weeks (closely comparable with adolescence to young adulthood in humans). The scientific data available show that constant variation in the field makes it considerably more active biologically.^41^

The results of our study do not support significant effects of cell phone EMR on visual and spatial memory, or anxiety level of “normal” mice. However, one should bear in mind that it is not possible to extrapolate these results into humans, hence, further studies are more than needed before reaching clear conclusions.

## Conclusion

The exposure of mice to cellular phone radiation in our study was brought about by placing the cellphones above the cages, not at all similar to the way humans use them (whole body exposure in mice vs. ear and half face exposure in humans). Further, the phones were on continuously for hours each day without a break, unlike the repeated intervals of phone calls during the day as occurs with people. In addition, the total daily exposure time in humans (especially in adolescents and young adults) may exceed the time of continuous exposure in mice. Hence, it is not a real-life condition of exposure. Another limitation is the notable difference between the brain structure and size of rodents and those of humans, imposing possible different target areas of involvement in the exposure process.

## Abbreviations Used

ANOVA: analysis of variance
BBB: blood–brain barrier
CCI: controlled cortical impact
CT: computed tomography
CTE: chronic traumatic encephalopathy
EMR: electromagnetic radiation
EPM: elevated plus maze
fMRI: functional magnetic resonance imaging
GCS: Glasgow Coma Scale
GSM: global system for mobile communication
LFP: lateral fluid-percussion
LSD: least significant difference
MRI: magnetic resonance imaging
mTBI: mild traumatic brain injury
MW: microwave
NOR: novel object recognition
PET: positron emission tomography
RF: radiofrequency
RF-EMR: radiofrequency electromagnetic radiation
RFR: radio frequency radiation
SD: standard deviation
SEM: standard error of the mean
SPECT: single photon computed emission tomography
TBI: traumatic brain injury
UMTS: Universal Mobile Telecommunications System
